# Crystal Structure of an Anti-Ang2 CrossFab Demonstrates Complete Structural and Functional Integrity of the Variable Domain

**DOI:** 10.1371/journal.pone.0061953

**Published:** 2013-04-17

**Authors:** Sebastian Fenn, Christian B. Schiller, Julia J. Griese, Harald Duerr, Sabine Imhof-Jung, Christian Gassner, Joerg Moelleken, Joerg Thomas Regula, Wolfgang Schaefer, Markus Thomas, Christian Klein, Karl-Peter Hopfner, Hubert Kettenberger

**Affiliations:** 1 Large Molecule Research, Pharma Research and Early Development (pRED), Roche Dignostics GmbH, Penzberg, Germany; 2 Discovery Oncology, Pharma Research and Early Development (pRED), Roche Diagnostics GmbH, Penzberg, Germany; 3 Discovery Oncology; Pharma Research and Early Development (pRED); Roche Glycart AG, Schlieren, Switzerland; 4 Department of Biochemistry, Gene Center, Ludwig Maximilians University Munich, Munich, Germany; University of Bologna & Italian Institute of Technology, Italy

## Abstract

Bispecific antibodies are considered as a promising class of future biotherapeutic molecules. They comprise binding specificities for two different antigens, which may provide additive or synergistic modes of action. There is a wide variety of design alternatives for such bispecific antibodies, including the “CrossMab” format. CrossMabs contain a domain crossover in one of the antigen-binding (Fab) parts, together with the “knobs-and-holes” approach, to enforce the correct assembly of four different polypeptide chains into an IgG-like bispecific antibody. We determined the crystal structure of a hAng-2-binding Fab in its crossed and uncrossed form and show that C_H_1-C_L_-domain crossover does not induce significant perturbations of the structure and has no detectable influence on target binding.

## Introduction

Therapeutic antibodies are used to treat a multitude of human diseases. They can routinely be obtained by mature technologies such as immunization or *in-vitro* display approaches. The natural variability of complimentarity-determining regions (CDRs) allows for the discovery of specific, high-affinity antibodies. Most of these therapeutic antibodies have the Immunoglobulin G (IgG) format which confers long serum half-life due to an FcRn-mediated recycling mechanism. In contrast to monospecific antibodies, bispecific antibodies offer additional features which cannot be accomplished otherwise, e.g. the selective targeting of a cell population characterized by two targets to improve safety and/or efficacy [1] [2].

A typical IgG antibody consists of two identical heavy chains (HCs) and two identical light chains (LCs) [3]. The N-terminal, antigen-binding domains of HCs and LCs are variable in sequence and are called V_H_ and V_L_ [4]. Typical IgG-type antibodies comprise two identical antigen-binding arms (Fabs), and an effector domain, Fc. Each Fab contains one light chain and heavy chain (reviewed by [5]).

The homodimerization of two HCs is achieved by strong non-covalent, predominantly hydrophobic interactions in the C_H_3-C_H_3 domain interface. In addition, HC homodimerization is stabilized by disulfide bridges in the lower hinge region. Unlike the C_H_3 domains, the C_H_2 domains are not involved in dimerization. Practically no protein contacts exist between the two C_H_2 domains of an IgG, but N-linked carbohydrates fill the intervening space. Instead, C_H_2 domains are responsible for the interaction with Fcγ receptors and the complement protein C1q [6].

Noteworthy, antibodies of the IgG4 subtype rapidly exchange half antibodies both *in vitro* and *in vivo* because the IgG4 hinge region allows for disulfide scrambling which breaks the covalent bonds between two HCs under redox-promoting conditions. Additionally, the C_H_3-C_H_3 domain interface provides weaker non-covalent contacts than in other IgG subtypes [7], [8].

The covalent HC-LC heterodimerization is achieved by a disulfide bridge between the C_H_1 and C_L_ domains. Additionally, strong non-covalent interactions between the V_H_ and V_L_ domains, and between the C_H_1 and C_L_ domains, respectively, enforce HC-LC pairing. The strength of V_H_ and V_L_ domain interaction, as well as the stability of the resulting V_H_V_L_ pair is influenced by germline family (reviewed in [9]) and CDR sequences [10]. Albeit HCs of any V_H_ germline family can stably interact with LCs of any V_L_ germline family, the exact factors that govern the stability of V_H_ and V_L_ domain interaction seem to be complex and still lack a mechanistic understanding [9]–[12]. The free C_H_1 domain is intrinsically disordered and was found to be stabilized by the interaction with the C_L_ domain. A molecular chaperone, BiP, binds to incompletely folded C_H_1 domains before it is replaced by the C_L_ domain. Additionally, a conserved proline residue undergoes isomerization during the C_H_1 folding process [13].

*In vivo*, every antibody-producing cell (e.g. B-cell) produces only one sort of antibody at a given time. Therefore there was no evolutionary necessity for preferential HC-LC association within a mixture of HCs and LCs. Consequently, co-expression of two different HCs and two different LCs, i.e. the constituents of two different antibodies, as observed in the “quadroma approach”, leads to a stochastic mixture of 10 different antibodies, in which the desired bispecific antibody is expected only in low amounts ([14], [15] and Figure S10 in [16]). Heterodimeric HC association can be achieved with high selectivity by the knobs-into-holes approach [17]–[20]. Here, residues in the C_H_3-C_H_3 interface are replaced by different residues in either heavy chain so that an asymmetric, mutually exclusive dimerization interface is formed. Heterodimers can be additionally stabilized by a disulfide bridge in the C_H_3 domain which is designed to form in heterodimers but not in homodimers.

Such HC heterodimers still associate with two different LCs in a non-selective way. One way to bypass this challenge is the use of a “common light chain” which is selected to provide – in combination with either of the HCs – high affinity binding to two different targets [21]. Creating selective, high-affinity antibodies sharing a common light chain however requires specific antibody generation approaches (e.g. by phage display) and is not readily applicable for the combination of two existing antibodies into a bispecific antibody.

**Figure 1:**
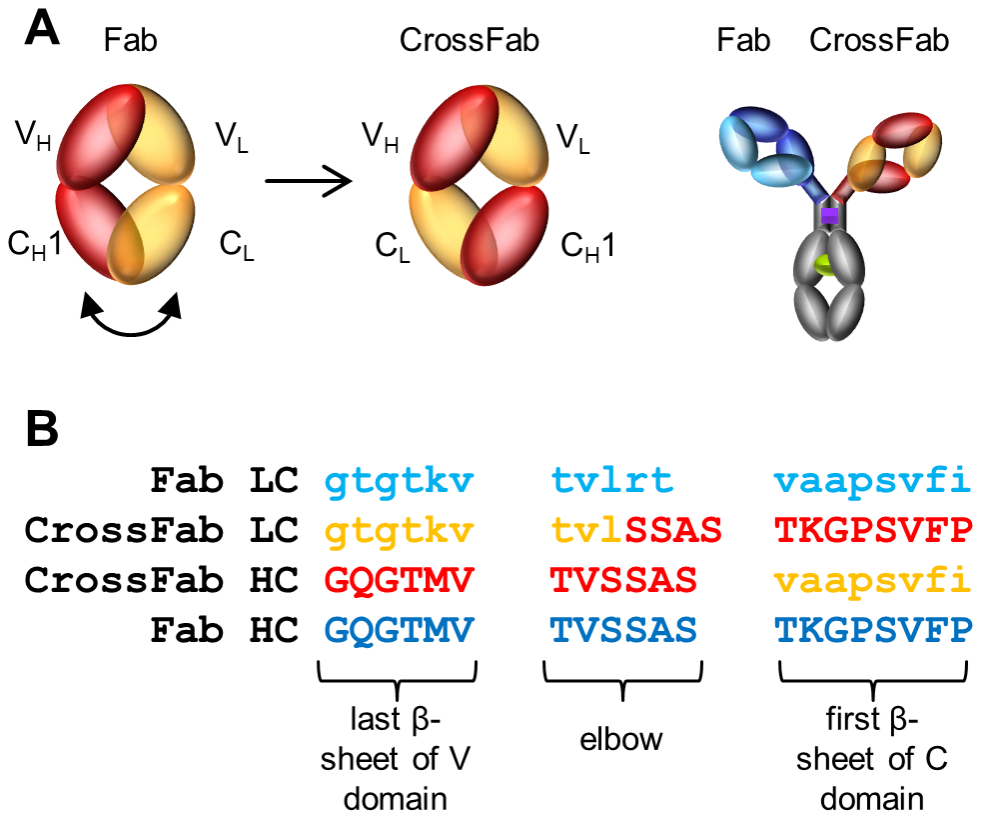
CrossMab design. (A) Schematic representation of the domain crossover leading to CrossFabs. Right: Combination of a Fab and a CrossFab to obtain a CrossMab [16]. Antibody domains are symbolized as ovals. Light colors are used for LC domains; darker colors are used for HC domains. This color code is used throughout. (B) Sequence alignment of the elbow and adjacent regions of LC and HC in Fab and CrossFab.

A generic approach to assemble two different heavy and two different light chains into a bivalent, bispecific IgG antibody (“CrossMab”) without artificial linkers was recently reported [16]. Correct pairing of two different LCs with their respective HCs is achieved by a C_H_1-C_L_-domain crossover in one of the Fabs (Figure 1A). In contrast to other approaches to generate bispecific IgG-like antibodies (e.g. reviewed in [1]), any existing pair of monoclonal antibodies can be combined into a CrossMab. This approach is based on the assumption that the overall structure of such a CrossMab closely resembles a normal IgG and that the Fab domains are not significantly altered compared to their uncrossed counterparts.

To reveal potential structural effects of the crossover procedure on the resulting bispecific antibodies, we report the crystal structure of a human Angiopoietin 2 (hAng2) binding “CrossFab”, i.e. a Fab derivative in which the V_L_ domain is fused to the C_H_1 domain, while the V_H_ domain is fused to the C_L_ domain (“C_H_1-C_L_ crossover” in [16]). For comparison, we also determined the crystal structure of the corresponding “uncrossed” Fab. Both structures show a high degree of similarity in the variable and constant domains. However, marked differences in their elbow angles (i.e. the angle between V_H_V_L_ and C_H_1CL domains, see Materials and Methods) are observed, which do not influence target binding.

## Results

### CrossFab design

The original, uncrossed Ang2-binding Fab was obtained by phage display of a scFv library and belonged to the IgG1λ subgroup. The uncrossed Fab was generated by adding constant domains C_H_1 and a C_L_ domain of the kappa subtype, which is the more frequently occurring light chain subtype in therapeutic antibodies.

For the design of the elbow crossing points, the X-ray structure 3NPS [22] was selected as a template because both chains of this Fab exhibit a high degree of homology with the anti-Ang2 Fab chains. In addition, both molecules possess a λ variable and a κ constant domain, and thus contain almost identical elbow sequences (Figure 1B).

Selection of the elbow crossing points aimed at maximal preservation of the native elbow structure, together with maximum sequence conservation to the native elbow sequences. Thus, structurally homologous elbow residues in the LC and the HC were identified by a structure-guided alignment of C_H_1 with C_L_ sequences, including the elbow regions, using the structure 3NPS as a template. Since the lengths of the HC and LC elbow sequences differ by one amino acid, the longer alternative was chosen on both the crossed LC and the crossed HC to avoid steric constraints. Extra care was taken to preserve the orientation of the side chain vectors Cα-Cβ in the crossed elbow region. A high degree of local sequence homology between the V_H_-C_H_1 and V_L_-C_L_ elbow regions allows construction of crossed elbow regions with a minimum number of non-conservative mutations.

An automated algorithm to detect potential T-cell epitopes did not show any alerts for the heavy chain-light chain transition sequences [23]. For purification and assay purposes, the Fab and CrossFab were expressed with a C-terminal Avi-His tag. Beside the C_H_1-C_L_ domain exchange, there are no further differences between the Fab and the CrossFab sequences.

### Crystal Structure

**Figure 2:**
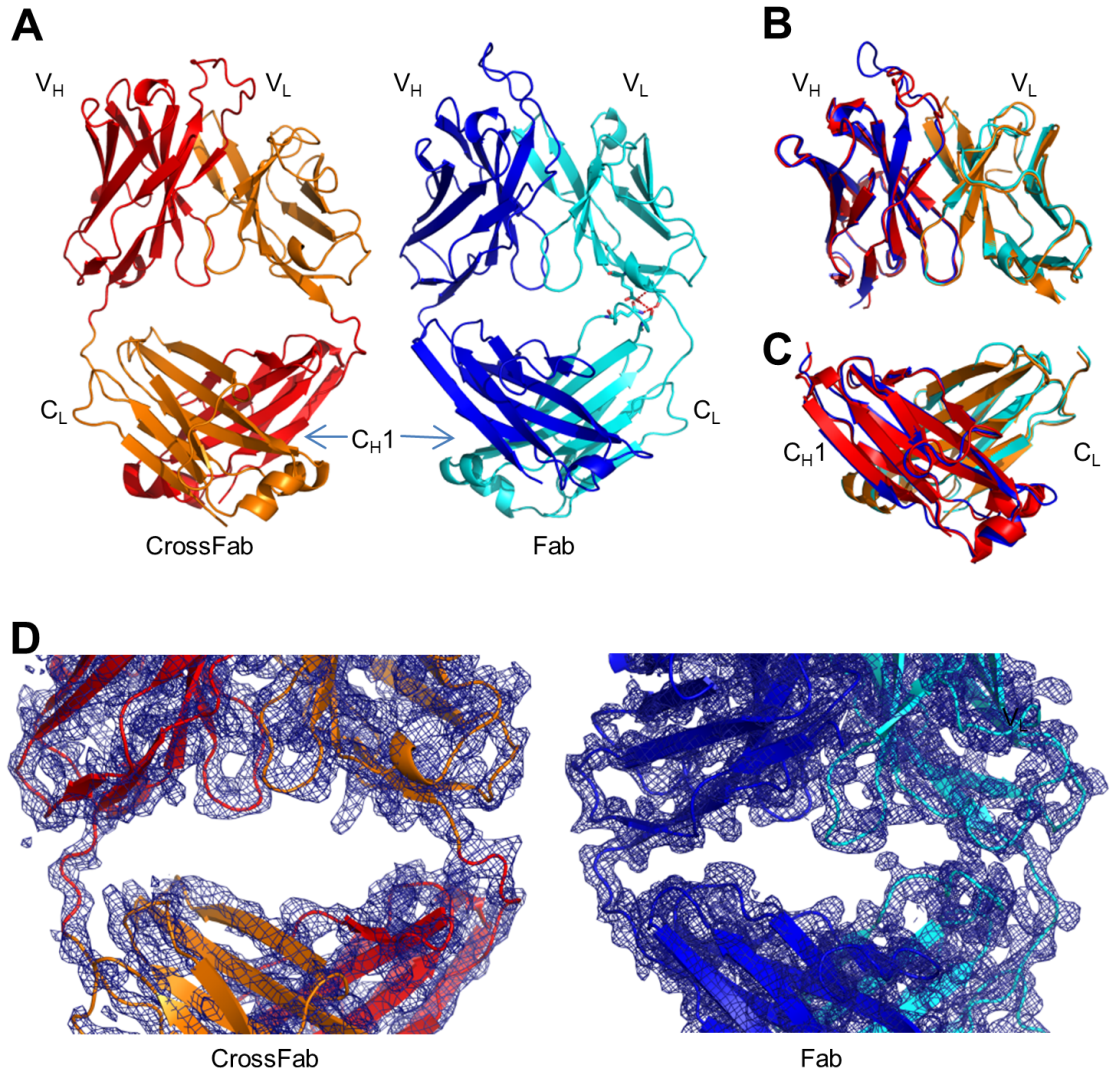
Crystal structures and electron density maps. (A) Comparison of the Fab and CrossFab structures side-by-side. Residues involved in hydrogen bonds between the constant and the variable domains are shown as sticks and hydrogen bonds are shown as red dashed lines. The color code used is as in Figure 1. (B) Superposition of variable domains in Fab and CrossFab. C-alpha atoms excluding CDR H3 were used. Fab and CrossFab variable domains superimpose with an rmsd of 0.43 Å. (C) Superposition of the constant domains in Fab and CrossFab using C-alpha atoms. Fab and CrossFab constant domains superimpose with an rmsd of 0.40 Å. (D) Composite omit maps around the V_H_V_L_-CH_1_C_L_ interface of the CrossFab and the Fab, contoured at 1.0 sigma using a carve distance of 2.0 Å.

The crystal structures of Fab and CrossFab, determined at a resolution of 2.2 and 2.9 Å, respectively, exhibit, as expected, the typical Fab geometry (Figure 2A). Although the diffraction data quality and hence the obtained resolution is slightly lower in the CrossFab (Table 1), the resulting electron density allowed unambiguous model building. The molecule regions directly affected by the CrossFab design, i.e. the elbow and adjacent regions, show clear electron density in unbiased composite omit maps, indicating a defined geometry and low flexibility (Figure 2D). The structural differences are most pronounced in loop regions (e.g. CDR 3 loops, Figure 2B) that intrinsically allow for a certain degree of flexibility in the absence of a bound antigen. A potential reason for the conformational difference between the heavy chain CDR3 loops from crystal structures of Fab and CrossFab may be the different role of this flexible loop element in mediating crystal-packing contacts in both structures. Nonetheless, the overall domain structure within the framework regions is very similar between Fab and CrossFab. A superposition of the V_H_ and V_L_ domains of Fab and CrossFab show a root mean square deviation (rmsd) of 0.43 Å (Cα atoms only, excluding CDR H3) (Figure 2B). The C_H_1 and C_L_ domains can be superimposed with an rmsd of the Cα atoms of 0.40 Å (Figure 2C), demonstrating the high structural similarity between the Fab and the CrossFab. The slight differences between domain structures of identical sequences in Fab and CrossFab may be explained by the different crystal packing, although both Fabs crystallized in space group P2_1_2_1_2_1_.

**Table 1:** Data collection and refinement statistics.

	CrossFab	Fab
**Data collection**		
Space group	P2_1_2_1_2_1_	P2_1_2_1_2_1_
Cell dimensions		
a, b, c (Å)	75.6, 80.6, 158.3	66.0, 86.7, 205.8
α, β, γ (°)	90.0, 90.0, 90.0	90.0, 90.0, 90.0
Resolution (Å)	45.26–2.93 (3.10–2.93)*	47.55–2.20 (2.33–2.20)
*R*_sym_ (%)^$^	17.3 (78.2)	4.1 (75.0)*
*I*/σ*I*	8.54 (2.09)	25.89 (2.72)
Completeness (%)	98.4 (90.4)	99.8 (98.8)
Redundancy	6.3 (6.0)	6.6 (6.7)
**Refinement**		
Resolution (Å)	44.10–2.93	46.76–2.20
No. reflections	21156	60561
*R*_work_/*R*_free_ (%)	23.7/26.9	20.0/22.6
No. atoms		
Protein	6724	6688
Ligand	12	18
Water	7	319
Average B-Factor	71.40	53.39
R.m.s. deviations:		
Bond lengths (Å)	0.003	0.004
Bond angles (°)	0.863	0.805

* Values in parentheses are for the highest-resolution shell. 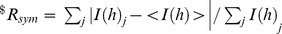, where 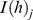 is the scaled observed intensity of the *j*th observation of reflection *h*, and 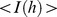
is the mean value of corresponding symmetry-related reflections.

**Figure 3:**
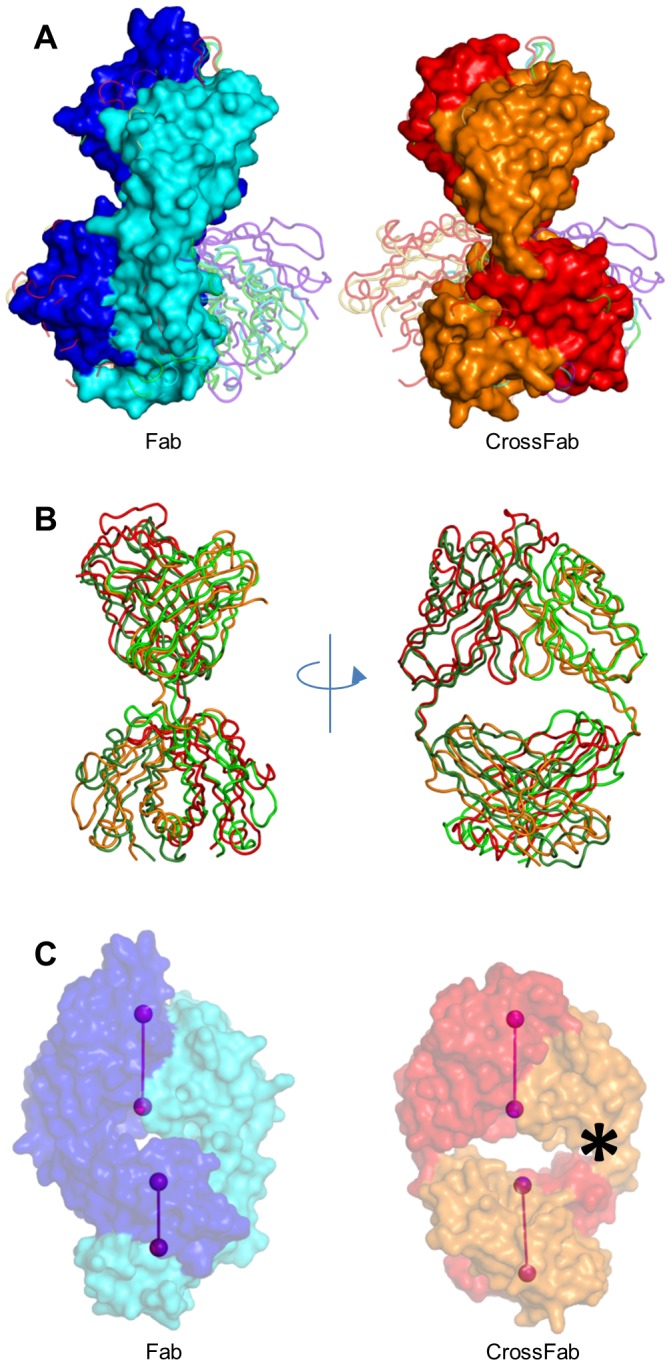
Elbow angles and relative domain orientation. (A) Superposition of Fab and CrossFab with example structures covering the range of observed elbow angles. Example structures (shown as loops), their orientations and color coding are according to Figure 1 in [24]. All structures are superimposed via their V_L_ domains. The colors of Fab and CrossFab are analogous to Figure 1. (B) Superposition of the CrossFab with its closest structural homolog (PDB code 3FMG). (C) Relative domain orientation of variable and constant domains in Fab and CrossFab. The pseudo-twofold axes of variable and constant domains are shown as light and dark purple dumbbells, respectively. Molecules are oriented so that the axes connecting the last Fv residues' C-alpha atoms, as well as the Fv pseudo-twofold axis are parallel to the paper plane. An asterisk marks the V_L_-C_H_1 junction, which is two amino acids longer than the corresponding V_L_-C_L_ junction.

The CrossFab structure shows clear electron density in the elbow region (Figure 2D), indicating that these engineered portions of the CrossFab are well-ordered. A striking difference of the Fab and CrossFab structures lies in the elbow angle, i.e. the angle between V_H_V_L_ and C_H_1C_L_ domains. The two molecules in the asymmetric unit of the Fab structure exhibit elbow angles of 138 and 145 degrees, respectively, whereas the CrossFab molecules in the asymmetric unit show 163 and 167 degrees, respectively. Like above, these differences may originate from differential crystal packing which may enforce distinct angles between the rather flexibly connected domains, although the artificial linker in the crossover procedure could have direct influence on the elbow angles or at least the conformational spectrum. A study of the distribution of elbow angles in experimental X-ray structures shows that possible elbow angles cover a wide range (127 to 220 degrees in the reported set of examples) [24]. Both elbow angles of the Fab and the CrossFab fall within this range (Figure 3A). To compare the overall structure of the CrossFab to published Fab structures, we used the "topsearch" program [25]. The closest match (PDB code 3FMG), was superimposed on the CrossFab (Figure 3B). It can clearly be seen that the domain orientation encountered in the CrossFab, especially the elbow angle, falls in the range exhibited by Fabs with natural domain organization. Most importantly, although one of the elbow chains contains one amino acid more than a native Fab, this does not cause a tilt between V_H_V_L_ and C_H_1C_L_ domains (Figure 3C). In summary, the structural analysis shows that the crossover procedure does result in a Fab that falls into the structural spectrum exhibited by the natural domain organization.

### Function and stability

**Figure 4:**
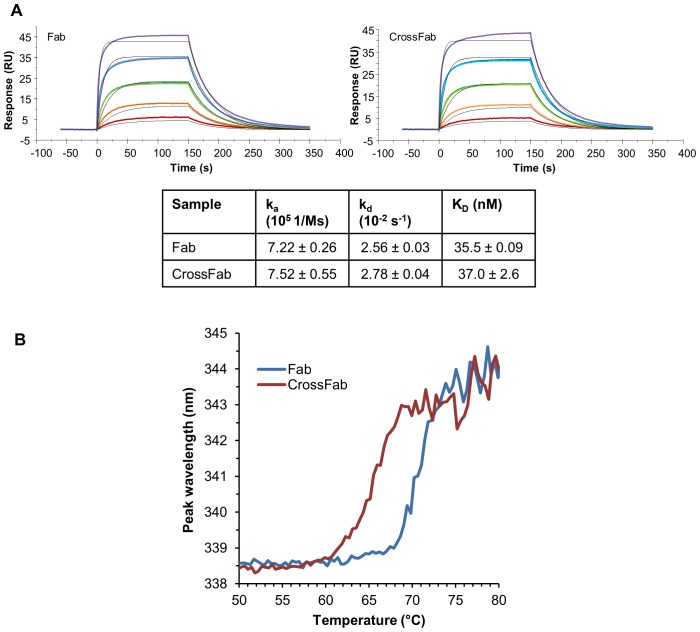
Target binding and thermal stability. (A) Surface plasmon resonance sensogram of the Fab and CrossFab interacting with their target, hAng2. One out of three runs for the Fab and the CrossFab is shown. Coloured curves represent the measured data at various Fab or CrossFab concentrations, while the result of the global fit to a 1∶1 Langmuir model is illustrated by black curves. The table shows average values and standard deviations derived from triplicate measurements (k_a_: association rate; k_d_: dissociation rate; K_D_: affinity). (B) Measurement of protein stability by temperature-dependent protein autofluorescence emission maximum wavelength.

To see whether CrossFab has target binding properties different from the uncrossed Fab we determined binding kinetics and affinity of the Fab and the CrossFab to their target, hAng2, by surface plasmon resonance. The measured affinities were 35.5±0.09 nM and 37.0±2.6 nM, respectively. Moreover, no significant difference in the kinetic parameters k_on_ and k_off_ was found, indicating that the structural features relevant for target binding are maintained in the CrossFab (Figure 4A).

Thermal stability of Fab and CrossFab was measured by temperature dependent intrinsic fluorescence since protein denaturation leads to a shift of the fluorescence emission maximum. Melting temperatures of 71°C and 66 °C were measured for the Fab and the CrossFab, respectively. (Figure 4B). The lower thermal stability of the CrossFab might be attributed to fewer stabilizing contacts between the variable and the constant domains in the CrossFab compared to the Fab. In essence, there are three pairs of residues forming hydrogen bonds between the V_L_ and C_L_ domains in the Fab but none such interactions were found in the CrossFab (Figure 2A). Nonetheless, the measured thermal stability of both Fab and CrossFab can be regarded as comparatively high [11], indicating no major structural perturbations.

## Discussion

In this report, we analyzed whether and to what extend the CrossMab design to generate bi-specific antibodies affects structural and target-binding properties of the crossed Fab region in comparison with the non-crossed parent Fab. CrossMab^CH1CL^ was shown to be expressed in eukaryotic cells with high fidelity concerning the correct HC-LC association [16]. This indicates a high degree of selectivity that originates both from the knobs-into-holes approach and the HC-LC crossover. In a CrossMab combining a crossed and an uncrossed Fab, all incorrect chain associations (e.g. an uncrossed HC with a crossed LC) involve domain contacts that are non-binding or even repulsive. Thus, sufficient selectivity is created to yield a relatively homogeneous product profile [16].

Taken together, these functional and structural data demonstrate the modularity of the immunoglobulin superfamily as applied to human antibodies. The data indicate that the CrossMab approach retains the structure of its parental antibodies and as such most if not all of their functional properties, thereby making it a generic approach that can be applied to any antibody pair. Because the CrossFab structure appears highly similar and unperturbed compared to the parental Fab structure, this may – together with the absence of any artificial linker sequences - be beneficial for low immunogenicity in humans.

Amongst the CrossMab alternatives published (CrossMab^CH1CL^, CrossMab^VHVL^ and CrossMab^Fab^), the CrossMab^CH1CL^ is preferred due to its theoretical side product profile that was confirmed when the different CrossMabs were expressed in parallel and analyzed for their side purity [16]. The approach chosen and in particular the elbow region in the CrossMab^CH1CL^ allows correct chain association and generation of a functional antibody as predicted. Based on our structural and biochemical data we believe that the CrossMAb approach represents a viable option for the generation of human bispecific heterodimeric IgG antibodies of different isotypes.

## Materials and Methods

### Cloning, expression and purification of Fab and CrossFAb

Fab and CrossFab constructs were designed to bear a C-terminal Avi-His_6_-tag at the C_H_1 domain. All genes were obtained via gene synthesis and cloned via unique restriction sites using standard cloning procedures. Every chain was part of a separate expression vector enabling secretory expression in human embryonic kidney (HEK) cells growing in suspension. Transfection into HEK293-F cells (Invitrogen) was performed according to the cell supplier's instructions using Maxiprep (Qiagen) preparations of the antibody vectors, Opti-MEM® I medium (Invitrogen, USA), 293fectin™ (Invitrogen, Germany) and an initial cell density of 1–2 million viable cells/ml in serum free FreeStyle 293 expression medium (Invitrogen). Cell culture supernatants were harvested after 7 days of cultivation in shake flasks or stirred fermenters by centrifugation at 14000 g for 30 minutes and filtered through a 0.22 µm filter.

Fab and CrossFab-containing supernatants were applied on a HisTrap column (GE Healthcare), washed with 20 mM sodium phosphate, 500 mM NaCl, 5 mM imidazole, pH 7.4 and eluted with washing buffer supplemented with 500 mM imidazole. Aggregated protein was removed by size exclusion chromatography (Superdex 200, GE Healthcare) in 20 mM Histidine, 140 mM NaCl pH 6.0. Monomeric protein fractions were pooled, concentrated if required using an Amicon Ultra (10 kD molecular weight cutoff) centrifugal concentrator (Millipore) and stored at −80°C. Purity was assessed to be >95% by SDS-PAGE and analytical size-exclusion chromatography.

### Cloning, expression and purification of hAng2

His-tagged full-length Ang2 was cloned by standard protocols and transiently expressed in HEK cells. Ang2-containing supernatant was applied to a HisTrap column (GE Healthcare) equilibrated with 20 mM sodium dihydrogenphosphate, 500 mM NaCl pH 7.4, washed with equilibration buffer supplemented with 20 mM imidazole and eluted in 20 mM sodium dihydrogenphosphate, 500 mM NaCl, 500 mM imidazole, pH 7.4. Full-length Ang2-containing fractions were pooled and dialysed against a 100-fold volume of dialysis buffer (20 mM Tris, 200 mM NaCl, 0.01% Tween 20, pH 7.5) overnight using slide-a-lyser dialysis cassettes (Thermo Scientific).

### Crystallization and structure determination

Prior to crystallization, both the Fab and the CrossFab were freshly thawed and applied to a Superdex 200 26/60 pg size exclusion column (GE Healthcare). Chromatography was performed at 8 °C in 20 mM HEPES-HCl, pH 7.0 and 150 mM NaCl for the Fab and in 20 mM Imidazole-HCl, pH 6.0 and 100 mM NaCl for the CrossFab. The peak fractions were pooled and concentrated immediately prior to crystallisation setups to 11 mg/mL in case of the Fab and 17 mg/mL in case of the crossed Fab using an Amicon Ultra (10 kD molecular weight cutoff) centrifugal concentrator (Millipore).

Both proteins were crystallized by the hanging drop vapor diffusion method at 20 °C. A volume of 1 µl of protein was mixed with 1 µl of reservoir solution (15%(w/v) PEG4000, 0.1 M Tri-Na-Citrate, 15%(v/v) Isopropanol in case of the Fab, and 17.5–20%(w/v) PEG 6000, 0.1 M citric acid, 0.2 M NDSB-221, pH 5.0, in case of the CrossFab). Crystals were cryoprotected by soaking them for 30 seconds in mother liquor solution containing 20%(v/v) glycerol, and flash frozen in liquid nitrogen. Diffraction data from single crystals were collected at 100 K at the beamline PX I of the SLS (Villigen, Switzerland).

### Data refinement and structure solution

Diffraction data were integrated and scaled with XDS [26] (see Table 1 for data collection and refinement statistics). In case of the CrossFab, the space group was determined by the program “pointless” [27] to be P2_1_2_1_2_1_, with two CrossFab molecules in the asymmetric unit [28]. The structure of the CrossFab was solved by molecular replacement with Phaser [29] using a polyalanine search model which was generated from a homology model of the CrossFab. The homology model itself was created using the program modeller 9v7 [30]. A highly homologous Fab structure (PDB code 3LMJ) was used as the modeling template. The final structure of the CrossFab was generated by multiple cycles of manual model building using COOT [31] followed by refinement using phenix refine [32]. The final R-factors of the model are R_work_ = 23.7% and R_free_ = 26.9%, respectively.

The structure of the uncrossed Fab was solved using Phaser [29] with the cross Fab structure as a replacement model. The variable domains and the constant domains were searched as separate models in Phaser. The model was completed by manual model building in the resulting electron density map using COOT [33]. The model was refined by iterative cycles of bulk solvent correction, individual B-factor refinement, translation-liberation-screw- and positional refinement using the programs Phenix [32] and Autobuster [34]. The final R-factors of the model are R_work_ = 20.0% and R_free_ = 22.6%, respectively.

Using the program CNS [35], cross-validated, sigma-A weighted 2F_o_–F_c_ composite omit maps were calculated to verify the accuracy of our structural models. For this we used the standard CNS protocol with a simulated annealing step from 500 K to 0 K.

According to [24], the elbow angle is defined as the angle between the pseudo-twofold axes between the light and heavy chain variable and constant domains, respectively. The elbow angle is the obtuse angle obtained by taking the arccos of the dot product of the two vectors. Elbow angles and pseudo-dyad axes were calculated using the PyMol script provided at http://www.pymolwiki.org/index.php/Elbow_angle. Intra-protein hydrogen bonds were detected using the PyMol function “find_pairs” with default settings. All figures of X-ray structures were prepared with PyMOL (www.pymol.org).

### Thermal stability

Thermal stability was measured using an Optim1000 system (Avacta Group plc) as the change in intrinsic protein fluorescence upon excitation at 266 nm. In a micro cuvette array, 9 µL of the samples in 20 mM Histidine, 140 mM NaCl, pH 6.0 at a concentration of 1 mg/mL were heated from 40 °C to 90 °C at a rate of 0.1 °C/min. Fluorescence emission spectra were recorded every 0.4 °C andprocessed with the software IgorPro, Version 6.23 (Avacta Group plc) in order to obtain the fluorescence emission peak wavelength. The melting point is defined as the inflection point of a peak wavelength versus temperature plot.

### Target binding assay

Surface Plasmon Resonance (SPR) experiments were performed on a BiacoreT200 instrument (GE Healthcare) at 25 °C using HBS-P (10 mM HEPES, 150 mM NaCl, 0.05% P20 pH 7.4) as running and dilution buffer. Full-length Angiopoietin-2 was immobilized on the surface of a CM5 sensor chip using standard amine-coupling chemistry yielding a surface density of approximately 500 RU. Three independent concentration series of the Fab and CrossFab, spanning a range between 300 and 3.7 nM, were injected. Two concentrations in each series were run in duplicate at the start and the end of a measurement series to proof surface stability. Association and dissociation time was 3 minutes with a flow rate of 50 µL/min. A regeneration solution of 10 mM sodium hydroxide was injected for 1 min at 5 µl/min flow rate to remove any non-covalently bound protein after each binding cycle. The experimental curves were fitted globally to a 1∶1 Langmuir binding model using the BIAevaluation software. The reported association, dissociation and affinity constants represent the average of these triplicate measurements, together with their standard deviations.

### Accession codes

Coordinates and structure factors were deposited at the Protein Data Bank (PDB) with accession numbers 4IMK (Fab) and 4IML (CrossFab).
